# Case study: Imperial College Healthcare NHS Trust

**Published:** 2019-09-10

**Authors:** Ali Mearza

**Affiliations:** 1 Consultant Ophthalmic Surgeon & Clinical Director, Imperial College Healthcare NHS Trust

The eye department at Imperial College Healthcare NHS Trust underwent an external review by the Royal College of Ophthalmologists, UK in January 2017.

The review was commissioned by the Medical Director of the Trust following a series of Never Events within ophthalmology in the preceding 18 months. It was felt at the time that an external review would be a positive step in highlighting areas of weakness as well as providing advice on improvements.

There was a change in leadership structure in July 2016, when a newly appointed clinical director and general manager joined the Trust. It was felt the review would be helpful in providing some direction to the new management team.

The review took place in January 2017. The process was very thorough, with information gathering beforehand and then detailed interviews with relevant members of staff as well as a detailed tour of the department.

Our main concern was the perception that the review would mark us down as a department and would only highlight the weak areas, but this was not true. The review was extremely helpful, in that it highlighted good practice and confirmed that the changes the new management team had already put in place were taking us in the right direction.

The review also highlighted limitations faced by our department, including issues with the building, IT infrastructure, nursing and admin structures. This aspect of the review results helped us to advocate for support for the changes we needed in order to do better.

For us, it was a very useful exercise as it highlighted good practice, identified areas of improvement, gave us ideas for some innovative developments within the department based on other's experiences and, importantly, gave us external validation that the department was safe and providing a good service, despite the constraints we faced.

**“Our main worry was that the review would only highlight our weak areas, but this was not true.”**

Two years on, and we are pleased to say that our structures are better, our department is stronger and better staffed, staff morale is higher and we have not had any Never Events since the original series that triggered the review in the first place.

We would recommend an external review to others. The review sets standards for what we should be doing and to what standard – based on what would be considered normal practice elsewhere – and it does this from an external viewpoint. An external review can be a powerful tool to advocate for, and implement, positive change.

